# Prevalence and epidemiologic features of adult upper gastrointestinal Crohn’s disease over the last 30 years: a scoping review

**DOI:** 10.1093/ecco-jcc/jjag168

**Published:** 2026-10-30

**Authors:** Daniel Lupas, Navneet Natt, Reyad Elzaanoun, Yuhong Yuan, Prince Taylor, Rokhsana Mortuza, Sudheer Vuyyuru, Jurij Hanzel, Christopher Ma, Eileen Crowley, Neeraj Narula, Silvio Danese, Laurent Peyrin-Biroulet, Siddharth Singh, Vipul Jairath, Rocio Sedano

**Affiliations:** Schulich School of Medicine & Dentistry, Western University, London, ON, Canada; Department of Medicine Division of Gastroenterology, Western University, London, ON, Canada; Schulich School of Medicine & Dentistry, Western University, London, ON, Canada; Department of Medicine Division of Gastroenterology, Western University, London, ON, Canada; Lawson Health Research Institute, London, ON, Canada; Schulich School of Medicine & Dentistry, Western University, London, ON, Canada; Temerty Faculty of Medicine, University of Toronto, Toronto, ON, Canada; Lawson Health Research Institute, London, ON, Canada; Department of Medicine Division of Gastroenterology, Western University, London, ON, Canada; Department of Gastroenterology, Faculty of Medicine, University of Ljubljana, Ljubljana, Slovenia; Division of Gastroenterology & Hepatology, Department of Medicine, Cumming School of Medicine, University of Calgary, Calgary, Alberta, Canada; Department of Community Health Sciences, University of Calgary, Calgary, Alberta, Canada; Division of Pediatric Gastroenterology, Department of Pediatrics, Children’s Hospital Western Ontario, Western University, London, Ontario, Canada; Division of Gastroenterology, Department of Medicine, Farncombe Family Digestive Health Research Institute, McMaster University, Hamilton, Ontario, Canada; Gastroenterology and Endoscopy, IRCCS Ospedale San Raffaele and University Vita-Salute San Raffaele, Milano, Italy; Division of Gastroenterology and Hepatology, McGill University Health Centre, Montreal, Quebec, Canada; Department of Gastroenterology, CHRU Nancy, INSERM NGERE, Université de Lorraine, Vandœuvre-lès-Nancy, France; Division of Gastroenterology and Hepatology, Department of Medicine, Mayo Clinic Arizona, Scottsdale, AZ, United States; Department of Medicine Division of Gastroenterology, Western University, London, ON, Canada; Lawson Health Research Institute, London, ON, Canada; Department of Epidemiology and Biostatistics, Western University, London, ON, Canada; Department of Medicine Division of Gastroenterology, Western University, London, ON, Canada; Lawson Health Research Institute, London, ON, Canada; Department of Epidemiology and Biostatistics, Western University, London, ON, Canada

**Keywords:** Crohn’s disease, upper gastrointestinal involvement, prevalence, epidemiology, scoping review

## Abstract

**Background:**

Crohn’s disease (CD) is an inflammatory bowel disease (IBD) that can affect any part of the gastrointestinal tract. Although upper gastrointestinal (UGI) manifestations are well recognized in pediatrics, UGI-CD in adults has historically been underestimated. Methodological limitations, including selective endoscopy and inconsistent classification, have contributed to variable prevalence estimates.

**Aims:**

Our goals were to summarize the prevalence of UGI-CD in adults with CD, understand demographic variations, and summarize knowledge gaps.

**Methods:**

We conducted a scoping review of MEDLINE, Embase, and Cochrane CENTRAL (1995–November 2025) using PRISMA-ScR and JBI methodology. Eligible studies reported adult UGI-CD prevalence or related epidemiologic data. Extracted variables included prevalence estimates, site-level involvement, and demographics. Systematic reviews were included to summarize review-level epidemiologic evidence.

**Results:**

In total, 117 primary studies and eight systematic reviews were included. Across 95 cohorts with prevalence data, the median prevalence of UGI-CD was 8.8% (IQR 3.99%-14.5%). Higher rates were observed in cohorts using systematic endoscopy and biopsy, while population-based cohorts reported lower values. Isolated UGI-CD was rare, with a median prevalence of 1.37% (IQR 0.87%-3.02%). Prevalence varied geographically and ethnically, with lowest rates in South Asian cohorts and higher rates in East Asian and Middle Eastern cohorts. Site-specific reporting was inconsistent, and few studies detailed modifiers such as smoking or family history.

**Conclusions:**

Reported prevalence estimates of adult UGI-CD remain highly variable due to inconsistent definitions and detection strategies. Standardized, site-specific adult definitions and systematic diagnostic protocols are needed to enable cross-study comparisons and thus improve prevalence estimates and address demographic reporting gaps.

## 1. Introduction

Crohn’s disease (CD) is a chronic relapsing inflammatory bowel disease (IBD) characterized by transmural inflammation, which can involve any part of the gastrointestinal tract.^1^ Although the terminal ileum and colon are the most commonly affected regions, involvement of the upper gastrointestinal (UGI) tract, including esophagus, stomach, duodenum, and jejunum, has been increasingly recognized.^2^ However, UGI involvement has been historically underestimated for several reasons, including selective endoscopy practices and the attribution of UGI lesions to other conditions such as *Helicobacter pylori* infection, nonsteroidal anti-inflammatory drug use, or nonspecific gastritis.^3^^,^^4^ Unlike pediatrics, the UGI tract is not routinely surveyed in adult patients with established IBD in the absence of symptoms, further leading to under-recognition of this entity. Consequently, estimates of adult UGI‑CD prevalence vary widely across cohorts, reflecting differences in case definitions and diagnostic pathways.

This under-recognition highlights the need for formal classification systems that could explicitly account for UGI disease. The Vienna classification first introduced “L4” as a designation for UGI disease, alongside the behavioral subtypes “B1” (non-stricturing non-penetrating), “B2” (stricturing), “B3” (penetrating), and “p” (perianal modifier).^5^ However, in this system, L4 served as a mutually exclusive category from other sites of CD involvement. This was refined in the Montreal classification, where L4 was redefined as a modifier that could coexist with ileal (L1), colonic (L2), or ileocolonic (L3) disease.^6^ In pediatrics, the Paris classification further subdivides L4 into L4a (esophagogastroduodenal) and L4b (proximal small bowel).^7^ Unfortunately, adult studies inconsistently apply these taxonomies, and reporting by area of UGI involvement (esophageal, gastric, duodenal, jejunal) is frequently incomplete, limiting comparability across cohorts.

Recent systematic reviews and meta-analyses have attempted to better characterize UGI-CD, with pooled prevalence estimates of approximately 13% and 15% reported in two large analyses,^8^^,^^9^ with higher estimates observed in studies incorporating systematic endoscopic evaluation. More recently, Cohen et al. reported a median adult UGI-CD prevalence of 8.7% (interquartile range [IQR] 4.74%-24.36%), with higher estimates observed when endoscopy and histology were performed systematically.^10^ However, prevalence estimates remain highly variable because of inconsistent definitions and diagnostic approaches, while some earlier analyses also included pediatric or mixed-age cohorts. In parallel, other studies have focused on the clinical implications of UGI involvement, suggesting associations with more aggressive disease behavior, including stricturing and penetrating phenotypes and an increased need for surgery.^8^^,^^9^^,^^11^ While some studies have explored geographic and ethnic variation in UGI-CD, findings remain inconsistent and are often limited by incomplete reporting of demographic variables.^12^^,^^13^ Furthermore, most prior literature does not clearly distinguish between isolated UGI disease and UGI involvement occurring alongside ileocolonic disease, with several reviews noting the rarity of isolated disease but lacking consistent definitions or standardized reporting.^8^^,^^14^

Despite advancements in the understanding of UGI involvement in CD, the epidemiology of UGI-CD remains incompletely characterized, particularly in adult populations. Given the limitations in the current literature, we conducted a scoping review that complements existing work by comprehensively describing the prevalence and epidemiologic features of adult UGI-CD, including assessment of geographic and ethnic variation, quantification of isolated UGI-CD, and identification of gaps in demographic reporting. Therefore, our objectives were to summarize reported prevalence ranges of UGI-CD, including esophageal, gastric, duodenal, jejunal, and general L4 classifications; to examine variation across age groups, geographic regions, and diagnostic practices; and to identify gaps in the literature that limit epidemiologic interpretation and cross-study comparability.

## 2. Methods

We performed a scoping review following the JBI scoping review methodology (https://jbi.global/scoping-review-network/resources) and the Preferred Reporting Items for Systematic Reviews and Meta‐analyses extension for Scoping Reviews (PRISMA‐ScR).^15^ The review question and eligibility criteria were developed using the Population–Concept–Context (PCC) framework recommended for scoping reviews.^16^ An in-house protocol was developed a priori to guide the design of this study; however, the protocol was not prospectively registered.

We conducted a literature search for full-text articles published in the English language using the MEDLINE, Embase, and Cochrane CENTRAL (via Ovid) databases from inception to November 26, 2025. The search strategy included keywords for CD and terms related to subsites of the UGI tract (Figure S1). It was designed to capture studies involving adult patients with CD and UGI involvement, with a focus on epidemiologic outcomes such as prevalence and disease characteristics. We limited inclusion to English‑language, adult or mixed‑age cohorts reporting extractable adult data (≥18 years). We excluded studies published prior to 1995 to better reflect contemporary diagnostic practices in the era of standardized reporting systems such as the Vienna and Montreal classifications.^5^^,^^6^ It also differs from earlier reviews that incorporated substantially older data, allowing assessment of whether more recent studies show different epidemiologic patterns. We excluded conference abstracts, case reports, trial registry records, animal studies, and studies that did not present primary research data such as narrative reviews or editorials. Systematic reviews and meta-analyses were included in this review, and references were checked, but the data presented were summarized separately and not extracted for primary prevalence data in order to avoid repetition of datapoints.

Titles and abstracts were screened independently by two reviewers (DL and NN) using Covidence.^17^ Articles deemed potentially eligible underwent full-text screening by two reviewers independently (DL and NN). Any discrepancies between reviewers during screening were resolved through discussion or, if necessary, adjudication by a third reviewer (YY or RS). At the title and abstract screening stage, records were eligible if they explicitly referenced UGI involvement in CD. At the full-text stage, studies were included for data extraction if they reported either the number or percentage of individuals in the cohort with UGI involvement or provided other epidemiologic data pertaining to UGI-CD. To minimize denominator heterogeneity, we preferentially extracted numerators/denominators where UGI involvement was reported against the total CD cohort at diagnosis or at a clearly defined timepoint. Studies reporting solely UGI‑only cohorts were not pooled in prevalence summaries. Studies were included if they reported adult cohorts or provided a mean or median cohort age of 18 years or greater. If studies included separate data for both adult and pediatric cohorts, the adult data were eligible for extraction. Pediatric-only data are presented in a separate study being conducted by our research team. For mixed-age cohorts, adult-specific data were extracted where reported; if only aggregate data were available without adult–pediatric separation, the studies were excluded from quantitative prevalence summaries but could be narratively described. Studies were also excluded if the UGI data included ileal involvement. Reference lists of relevant studies were screened, and additional articles identified by the research team were also included.

Data extraction was conducted using a standardized form which was developed a priori and piloted on a subset of studies before use. Data were extracted independently by one reviewer (DL) and verified by three senior team members (NN, YY, and RS), with corrections made through consensus. Extracted fields included study identifiers, study design, country, study duration, total population with IBD and CD, respectively, number of individuals with UGI involvement (with subsite details when available), age and sex distributions, ethnicity, smoking status, family history of IBD, extraintestinal manifestations, disease behavior, and study-specific main takeaways and limitations. Critical appraisal of individual study quality was not performed. No meta-analysis was performed due to heterogeneity of UGI-CD definitions, study populations, and diagnostic practices.

## 3. Results

### 3.1. Study selection and characteristics

In the initial literature search, 3455 records were identified, and one additional record was identified through peer review. In total, following the removal of 158 duplicates, 3298 records were screened at the title and abstract phase. Of these, 2565 were excluded, leaving 733 studies for full-text review. In the final scoping review, 125 studies were included, including 117 primary studies reporting prevalence or epidemiologic data and eight systematic reviews.^8-14^^,^^18-133^ The study selection process is summarized in Figure S2. We included 117 primary studies, and their key characteristics are detailed in Table 1. Similarly, the key characteristics, main findings, and limitations of the eight identified systematic reviews are summarized in Table 2.

**Table 1.** Summary of primary studies included in the scoping review (*n* = 117).

**Total studies included**	117
**Publication years**	1997-2025
**Geographic distribution (top contributors)**	United States (18), China (11), South Korea (9), Canada (8), Italy (6)
**Study designs**	Retrospective cohort (38), Retrospective case-control (6), Retrospective cross-sectional (7), Prospective cohort (6), Retrospective case series (4)
**Total CD patients across studies^a^**	78 683
**Total UGI-CD patients across studies^a^**	5011
**UGI-CD prevalence (median)^b^**	8.8% (IQR 3.99%-14.5%)
**Isolated UGI-CD prevalence (median)**	1.37% (IQR 0.87%-3.02%)
**Median age (total CD cohorts)^c^**	32.0 years (reported in 90 studies)
**Median age (UGI-CD subgroups)^c^**	27.0 years (reported in 16 studies)
**Sex (total CD cohorts)**	Female 49.7%, male 50.3% (reported in 103 studies)
**Sex (UGI-CD subgroups)**	Female 45.5%, male 54.5% (reported in 24 studies)
**UGI subsite reporting**	Most studies (103/117) reported only undifferentiated L4 involvement. Among those with site-level detail: duodenal cases ranged from 29% to 71% of UGI-CD cases (*n* = 175), gastric 43%-84% of UGI-CD cases (*n* = 172), esophageal 9%-29% of UGI-CD cases (*n* = 142), and jejunal-specific prevalence data were reported as 57% of UGI-CD cases (*n* = 72)

a Totals are cumulative across studies and may not represent unique patients due to potential cohort overlap.

b Prevalence calculated from general L4 only; isolated-L4 and subsite-only percentages excluded.

c For median age, study-reported medians were used when available, and means were substituted if medians were not provided.

Abbreviations: CD, Crohn’s disease; UGI, upper gastrointestinal; UGI-CD, upper gastrointestinal Crohn’s disease; IQR, interquartile range.

**Table 2.** Key characteristics of the systematic reviews included in the scoping review.

Author Year	Journal	Total studies/total patients	Main findings pertaining to upper gastrointestinal Crohn’s disease	Limitations
**Afzali 2016**	*Inflammatory Bowel Diseases*	47 studies/33 484 patients (20 054 African American; 10 762 Hispanic; 2668 Asian)	Upper gastrointestinal involvement was reported in African Americans in 11 studies (2%-27.4%). It was reported in Hispanics in six studies (0%-11.1%) and in Asians in one study (23.1%). This review provided pooled prevalence estimates for racial and ethnic minority populations in the United States.	Standard disease classifications (Montreal/Vienna) were not consistently applied. Reporting was aggregate rather than patient-level, and biological, endoscopic, and radiographic assessments were lacking.
**Amadu 2024**	*World Journal of Methodology*	9 studies/44 patients	This review focused specifically on duodenal Crohn’s disease. It reported that diagnostic delays were common and often led to complications. Endoscopy, radiology, and biopsy were often all required to establish the diagnosis.	The sample was very small and drawn largely from case reports, limiting generalizability. Diagnostic criteria were inconsistent across studies.
**Attauabi 2021**	*Therapeutic Advances in Gastroenterology*	23 studies/1572 CD with IMIDs and 35 043 CD without IMIDs	Patients with co-occurring IMIDs were more likely to have upper gastrointestinal involvement than those without IMIDs (RR 1.49, 95% CI 1.09-2.04). The review noted that L4 involvement in the included studies comprised both isolated and non-isolated disease.	Many studies did not provide specific L4 percentages despite inclusion in the meta-analysis, and under-reporting of individual IMIDs was likely.
**Avalos 2018**	*Digestive Diseases and Sciences*	7 studies/2273 patients (687 Hispanic; 1586 NHW)	Among patients with Crohn’s disease, Hispanics were less likely to have upper gastrointestinal involvement than non-Hispanic white patients (NHW), although this did not reach statistical significance (OR 0.58, 95% CI 0.32-1.06, *P* = .07). Overall anatomic distribution was similar, with a trend towards less upper GI disease in Hispanics.	Heterogeneity among cohorts was substantial and key modifiers such as migration status, duration in the USA, and ancestral background could not be accounted for, potentially masking subgroup differences.
**Chin 2021**	*Digestive and Liver Disease*	27 studies/10 853 patients	The pooled prevalence of UGI-CD was 13%. Studies that used endoscopy to define UGI-CD reported a higher prevalence of 23%. L4-jejunal disease was associated with the highest risk of surgery. Young male patients with erythema nodosum, aphthous ulcers, and stricturing disease were more likely to have UGI-CD, which was linked to increased hospitalization and surgery.	Heterogeneity was substantial across analyses (*I* ^2^ >75%). Africa and South America were under-represented, most studies were retrospective, and only a few evaluated risk factors in detail, limiting generalizability.
**Cohen 2025**	*Clinical Gastroenterology and Hepatology*	47 studies/1730 patients with UGI-CD. Prevalence analysis: 24 studies/6054 patients with CD, including 1010 with UGI-CD.	The median prevalence of UGI-CD was 8.7% (IQR 4.74%-24.36%), increasing to 28% with systematic endoscopy and 30.7% with systematic endoscopy and histologic assessment. Isolated UGI-CD accounted for 7.8% of UGI-CD cases, and 35.4% of patients were asymptomatic. Median ages were 23.1 years at initial CD diagnosis and 26.7 years at UGI-CD diagnosis; 43.8% were female, 21.2% had ever smoked, and 33.3% had EIMs among studies reporting each characteristic. The duodenum was the most common overall UGI location, and inflammatory and stricturing UGI behavior were each reported in approximately 55%. Anti-TNF therapy achieved an overall clinical response of 81%.	Although the overall risk of bias was assessed as low, most studies varied in their definitions, diagnostic approaches, and outcome measures. UGI-CD was restricted to the esophagus, stomach, and duodenum, excluding jejunal involvement included within broader Montreal L4 definitions. The included studies spanned 1976 to 2024, and changes in definitions and diagnostic methods over this period may have limited comparability across studies.
**Pauwels 2022**	*Clinical Gastroenterology and Hepatology*	14 studies/1317 patients	Upper gastrointestinal involvement (L4) was identified as one of several risk factors for relapse after anti-TNF cessation (HR 1.33, 95% CI 0.97-1.82). Other risk factors included smoking, younger age at diagnosis and cessation, absence of immunosuppressant use, elevated CRP, longer disease duration, and second-line anti-TNF therapy.	Eleven cohorts were excluded due to unavailable individual participant data. The model predicts relapse at 1 year and may not reflect longer-term outcomes. Missing biomarker data restricted inclusion of some clinical variables.
**Tamizifar 2023**	*JGH Open*	32 studies/14 509 patients	The pooled prevalence of UGI-CD was 15% (95% CI 11%-18%). The most prevalent behavior was B1 (non-stricturing–non-penetrating) at 38%. UGI-CD patients exhibited more stricturing disease (B2) than non-UGI-CD patients (0.38 vs 0.30, *P* = .03). UGI-CD was associated with a more aggressive course, including higher risks of surgery and drug therapy.	Most studies were retrospective, and no reliable temporal trend in UGI-CD prevalence could be established. Substantial heterogeneity was present across studies, and risk-factor analyses were limited.

Abbreviations: CD, Crohn’s disease; UGI/UGI-CD, upper gastrointestinal/upper gastrointestinal Crohn’s disease; L4, Montreal classification site for upper gastrointestinal involvement; B1/B2/B3, Montreal behavior categories (B1 inflammatory; B2 stricturing; B3 penetrating); IMIDs, immune-mediated inflammatory diseases; NHW, non-Hispanic white; anti-TNF, anti-tumor necrosis factor; RR/OR/HR, relative risk/odds ratio/hazard ratio; CI, confidence interval; CRP, C-reactive protein.

Of the 117 included primary studies, 44 were conducted in Europe and 30 were conducted in Asia. Prevalence of UGI-CD in North America was reported in 30 studies. Fewer studies were conducted in the Middle East (eg, Saudi Arabia, Israel, Turkey) and South America (eg, Brazil). Several multi-country and international collaborations were also included. Publication years of the included primary studies ranged from 1997 to 2025, with the majority published after 2010. Most included studies were retrospective in design, with approximately two-thirds classified as retrospective cohort or observational studies. Prospective cohorts accounted for 16 studies. Additional characteristics of the included studies are summarized in Table 1.

### 3.2. Prevalence of upper gastrointestinal Crohn’s disease

To calculate prevalence of UGI-CD, we analyzed data from 95 studies that included extractable prevalence data for UGI involvement in adult patients with CD. Across these 95 studies, the median rate of UGI involvement was 8.8% (IQR 3.99%-14.5%). The lowest prevalence (3% or lower) was noted in population-based studies or tightly defined hospital cohorts.^103-105^^,^^107^^,^^111^^,^^119^^,^^120^^,^^123^ Conversely, higher rates of UGI involvement (prevalence rates of 20% or greater) were found in studies where routine esophagogastroduodenoscopy (EGD) was performed on every patient, many of whom were asymptomatic.^2^^,^^3^^,^^34^^,^^60^^,^^62^^,^^114^ An example of unusually high reported rates comes from Matsumura et al. who found UGI involvement in 92.2% (83/90) of patients, likely reflecting systematic endoscopic screening, inclusion of minor lesions such as erosions and “bamboo joint-like” changes, and broader diagnostic practices.^62^ Additionally, when the same patients were assessed by different methods, prevalence values often changed dramatically. In one cohort, systematically performing endoscopy at the time of CD diagnosis revealed UGI involvement in 43.7% (227/520) of patients, with 68% (154/227) of these cases being asymptomatic.^114^ By contrast, a study that relied on computed tomography (CT) enterography (CTE) detected UGI disease in only 4.5% (10/221) of patients, and just 0.9% (2/221) had isolated UGI disease.^69^ Large multicenter cohorts tended to fall in the middle of the range, such as the Swiss IBD cohort, reporting UGI involvement in 6.5% (107/1638) of patients at diagnosis and 13.1% (214/1638) at follow-up.^43^ Notably, isolated UGI disease was much less common than UGI involvement with concomitant L1–L3 disease, with a median of 1.37% (IQR 0.87-3.02%) across 15 studies that provided data on isolated UGI-CD.^14^^,^^60^^,^^69^^,^^85^^,^^102^^,^^105^^,^^107^^,^^109^^,^^111-113^^,^^117^^,^^118^^,^^122^^,^^133^ To further illustrate the variability in reported prevalence estimates across geographic regions and study cohorts, a supplementary visual summary of individual study-level prevalence distributions is provided in Figure S3.

### 3.3. Geographic and ethnic variation in prevalence

The prevalence of UGI-CD varied by geographic location. South Asian studies generally reported the lowest prevalence, often below 5%.^21^^,^^31^^,^^93^^,^^116^^,^^128^ European and North American studies typically fell in an intermediate range of 5%-15%: for example, 11.7% (119/1015) and 12.9% (113/877) in two Canadian cohorts, respectively, and 13% (214/1638) in the Swiss IBD cohort.^40^^,^^41^^,^^43^ Higher prevalence estimates (15% or higher) were more commonly observed in Middle Eastern and East Asian cohorts, including Saudi Arabia (15.6% and 23.8%), China (17.7% and 22.7%), and South Korea (7%-20%).^3^^,^^19^^,^^24^^,^^26^^,^^48^^,^^51^^,^^54^^,^^55^ Although only 65 of 117 studies explicitly reported ethnicity, ethnic variation was also seen within countries. For example, a study in the United States revealed Black patients with CD were more likely than White patients to develop esophagogastroduodenal disease (20% [139/697] vs 8.5% [59/597]),^64^ while another U.S. cohort reported a higher prevalence among non-Hispanic Whites compared to Hispanics (12.5% vs 3.9%) (*P* < .05).^29^ A study in Israel revealed Ethiopian Jews had markedly higher prevalence than Ashkenazi Jews (23% vs 3%) (*P* < .016).^80^

### 3.4. Age and sex distribution of UGI-CD

Age was reported for the total IBD population in 90 of 117 studies (76.9%) and for the UGI-CD subgroup in 16 of 117 studies (13.7%). Reported ages were generally in the early thirties across studies with a median of 31.9 years (IQR 27-38) for the total IBD population, while the UGI-CD subgroup had a median of 27 years (IQR 23-32). Across studies that reported age in both individuals with UGI-CD and the overall IBD cohort, there were no significant differences in age between the two cohorts. Sex distribution was reported for the total population in 103 of 117 studies (88%) and for UGI-CD in 24 of 117 studies (20.5%). The median female proportion in the total IBD population was 49.7% (IQR 38.2-56.1). In UGI-CD, the median female proportion was 45.5% (IQR 31.8-58.5). In the 19 studies reporting sex distributions for total IBD and UGI-CD groups, there was a slight male predominance of UGI-CD, though differences were small and inconsistent.

### 3.5. Anatomical distribution of upper gastrointestinal involvement

Most of the primary studies reported only undifferentiated UGI involvement (L4) without further site-level detail. Among the studies that provided subsite prevalence data, duodenal involvement ranged from 29% to 71% of UGI-CD cases (*n* = 175). Gastric involvement ranged from 43% to 84% of UGI-CD cases (*n* = 172). Esophageal involvement was less common, ranging from 9% to 29% of UGI-CD cases (*n* = 142). Jejunal disease was typically reported together with proximal ileal data. In the single cohort providing jejunal-specific prevalence data, jejunal involvement was reported as 57% of UGI-CD cases (*n* = 72).^87^

### 3.6. Lifestyle and genetic factors associated with UGI-CD

Smoking status in UGI-CD was reported in only a small number of studies (*n* = 12). Reported proportions varied, with some cohorts describing very low rates and others higher frequencies, ranging from 0% (0/8) to 47.5% (96/202).^17^^,^^35^ Overall, the available evidence was inconsistent and insufficient to establish a clear relationship between smoking and UGI-CD.

A small number of cohorts addressed genetic associations. This included the absence of UGI involvement in individuals with the MIF -173G/C (C allele), IL23R p.381Gln, or carriers of the CCR5Δ32 mutation, or the significant association of the variant allele of the IL-12B gene (rs10045431) with UGI-CD.^37^^,^^45^^,^^65^

UGI-specific family history reporting was limited (11 studies), and most provided only crude proportions. Deng et al. found that familial CD cases showed a relatively high rate of UGI involvement (54%) (26/48), with good concordance for UGI involvement among family members.^34^ Conversely, another study found that UGI involvement was less prevalent in familial CD compared to sporadic cases, suggesting that family history may not be a predictor of UGI disease.^36^

### 3.7. Clinical phenotype in UGI-CD

Reporting of extraintestinal manifestations (EIMs) in patients with UGI-CD was limited to five studies.^26^^,^^43^^,^^78^^,^^81^^,^^85^ A Romanian study (*N* = 1099) found that UGI involvement was a significant independent risk factor for developing EIMs (univariate odds ratio [OR] 3.54, 95% confidence interval [CI] 2.87-5.64, *P* = .021; multivariate OR 4.90, 95% CI 2.27-5.96, *P* = .021).^81^ No consistent link between EIMs and UGI involvement was identified in the remaining four studies.^26^^,^^43^^,^^78^^,^^85^

Behavioral phenotype (B1/B2/B3/p) of CD in patients with UGI involvement was reported in 18 studies. Several cohorts specifically linked UGI-CD with more aggressive disease behavior, including stricturing (B2) and penetrating (B3) disease, and increased rates of surgery and hospitalization.^26^^,^^27^^,^^32^^,^^35^^,^^70^^,^^71^^,^^73^^,^^91^^,^^118^^,^^119^ However, these findings were not unanimous, as other studies did not identify a distinctive behavioral pattern or worse clinical outcomes in the L4 subgroup.^43^^,^^89^

### 3.8. Findings from systematic reviews

Beyond the primary literature, eight systematic reviews provided broader perspectives on UGI-CD, including review-level prevalence estimates and analyses of clinical and demographic factors. Tamizifar et al. conducted the largest meta-analysis, spanning 32 studies and 14 509 patients with a pooled prevalence of 15%. The authors also found that UGI-CD was associated with higher rates of stricturing phenotype, surgical resection, and a more aggressive disease course.^9^ This is similar to data presented in a systematic review by Chin et al. which identified an UGI-CD prevalence of 13%, though this value was as high as 23% when diagnosis was confirmed endoscopically across 27 studies.^8^

More recently, Cohen et al. conducted a systematic review focused specifically on adult UGI-CD, synthesizing 47 studies involving 1730 patients with UGI-CD and examining prevalence, clinical presentation, diagnosis, and management. Among the 24 studies contributing prevalence data (*N* = 6054; 1010 with UGI-CD), the median prevalence was 8.7% (IQR 4.74%-24.36%). Median prevalence increased to 28% in studies that performed endoscopy in all patients and 30.7% in those that systematically performed both endoscopy and histologic assessment. Cohen et al. also reported that 7.8% (81/1035) of patients with UGI-CD across 32 studies had disease isolated to the UGI tract and found that 35.4% (83/234) of patients with UGI-CD were asymptomatic across nine studies reporting this outcome. Cohen et al. also reported median ages of 23.1 years at initial CD diagnosis and 26.7 years at UGI-CD diagnosis, a median interval of 3.2 years between diagnoses, and proportions of 43.8% female, 21.2% ever-smokers, and 33.3% with EIMs among studies reporting each characteristic. Lastly, they also reported duodenal, gastric, and esophageal involvement in 71.8%, 57.4%, and 30.8% of UGI-CD cases, respectively. B1, B2, B3, and perianal phenotypes were reported in 44.4%, 38%, 19.4%, and 39.4%, respectively, while UGI stricturing and penetrating disease were reported in 55% and 6.3% among studies reporting each characteristic.^10^

Afzali et al. synthesized 47 studies (*N* = 33 484) of racial and ethnic minorities in the United States, reporting UGI prevalence of 2%-27% in African Americans, 0%-11% in Hispanics, and 23% in a single small Asian cohort.^12^ Avalos et al. also analyzed ethnic trends of UGI-CD in the USA and compared rates of UGI involvement in Hispanic versus non-Hispanic White patients. The authors observed a trend toward less UGI-CD involvement in Hispanics (OR 0.58), though not statistically significant.^13^ Attauabi et al. examined CD with co-occurring immune-mediated inflammatory diseases (IMIDs), reporting an increased risk of UGI involvement (relative risk [RR] 1.49).^131^ However, this association did not reach significance for any single IMID subgroup, such as primary sclerosis cholangitis, ankylosing spondylitis, or celiac disease. Pauwels et al. developed a prediction model for relapse after anti-tumor necrosis factor (anti-TNF) cessation, identifying L4 involvement as one of several risk factors for relapse.^11^

## 4. Discussion

This scoping review summarizes 30 years of adult UGI-CD epidemiology, revealing substantial variability in prevalence estimates across geographic regions and study designs. Among 117 primary studies, the median reported prevalence was 8.8% (IQR 3.99%-14.5%). Much of the variation was attributable to methodological approach. Population-based or retrospective chart review cohorts typically reported very low prevalence,^103-105^^,^^107^^,^^111^^,^^119^^,^^120^^,^^123^ whereas studies incorporating routine upper endoscopy and histopathology at diagnosis reported markedly higher estimates (40%-92%).^2^^,^^34^^,^^60^^,^^62^^,^^114^ Despite these differences, most large multicenter cohorts fell within a middle range of 5%-15%.^40^^,^^41^^,^^43^^,^^66^^,^^86^ Isolated UGI disease was largely uncommon in adults, with a median prevalence of 1.37% (IQR 0.87%-3.02%). In addition, geographic and ethnic variation was apparent. South Asian cohorts reported a much lower prevalence (5% or lower) in several studies. In contrast, most North American and European cohorts ranged between 5% and 15%, and Middle Eastern and East Asian studies frequently reported higher rates (greater than 15%).

Eight systematic reviews were included in this scoping review, providing broader context for interpreting the variability observed in the primary literature. Collectively, these reviews reinforce that UGI-CD is not rare, with two large meta-analyses reporting prevalence estimates of 13% and 15%.^8^^,^^9^ More recently, the comprehensive adult-specific systematic review by Cohen et al. reported a median prevalence of 8.7% (IQR 4.74%-24.36%), closely aligning with the median of 8.8% identified in our review.^10^ Cohen et al. also found that median prevalence increased to 28% with systematic endoscopy and 30.7% when both endoscopy and histologic assessment were performed systematically, further supporting our findings in regard to the influence of diagnostic intensity on prevalence estimates.^10^ Collectively, these reviews also suggest that UGI involvement may be associated with a more complicated phenotype, with higher rates of stricturing, surgery, and relapse after therapy withdrawal.^8-11^

Although the median prevalence identified in our review is broadly consistent with the existing systematic review literature and closely aligns with that reported by Cohen et al., our review and Cohen et al. address distinct but complementary questions. Cohen et al. combined an adult-specific prevalence synthesis with characterization of clinical presentation, endoscopic and histologic findings, and medical, endoscopic, and surgical management, using a definition restricted to the esophagus, stomach, and duodenum.^10^ In contrast, our scoping review maps UGI-CD prevalence and epidemiologic patterns across a larger body of primary studies, including studies reporting jejunal involvement and undifferentiated L4 disease, while restricting inclusion to studies published from 1995 onward to better reflect modern diagnostic practices and the emergence of standardized classification systems. Our review also specifically examines geographic and ethnic variation and gaps in demographic and site-specific reporting. Although both reviews quantified isolated UGI-CD, they addressed different questions: Cohen et al. found that 7.8% of patients with UGI-CD had disease isolated to the UGI tract, whereas our review estimated a median isolated UGI-CD prevalence of 1.37% within total adult CD cohorts. Cohen et al.’s finding that prevalence increased with systematic endoscopy and histologic assessment therefore complements the pattern of variation associated with diagnostic intensity observed across the primary studies in our review.^10^

The findings of this review may have important implications for clinical practice. Higher prevalence estimates in studies using systematic EGD, together with Cohen et al.’s finding that more than one-third of patients with UGI-CD were asymptomatic,^10^ suggest that UGI involvement may go unrecognized when evaluation is based on symptoms alone. Given that UGI involvement has been associated with more complicated disease phenotypes in multiple studies, earlier identification may be clinically relevant. These observations raise the question of whether a more systematic approach to UGI evaluation at the time of diagnosis should be considered. However, it remains unclear whether earlier identification meaningfully impacts clinical management or patient outcomes, and prospective studies are needed to better define the clinical relevance.

A key limitation across the literature was the inconsistency in how UGI involvement was defined and reported. Some studies limited UGI disease to the esophagus, stomach, and duodenum, while others extended definitions to the jejunum or even the proximal ileum. In several cohorts, UGI disease was reported only as “L4 involvement,” without clarification of whether this reflected isolated disease or coexistence with ileocolonic involvement. Some studies used the Montreal classification, while others used the Vienna system or no classification was specified at all. This lack of standardized definitions limits comparability across studies and contributes to the current knowledge gap in the reported prevalence and epidemiologic characterization of UGI-CD. Recent expert consensus efforts have sought to address this issue by proposing more standardized definitions and diagnostic criteria for UGI-CD.^134^ The variability observed across studies in our review reinforces the need for such standardization and highlights its importance for improving comparability and interpretation of epidemiologic findings across studies.

In addition, many studies did not explicitly report data regarding ethnicity. Where this information was available, important trends emerged, including higher prevalence of UGI-CD in Black patients compared with White patients in the United States, in non-Hispanic Whites compared with Hispanic patients, and in Ethiopian compared with Ashkenazi Jewish patients in Israel.^29^^,^^64^^,^^80^ Nonetheless, further research is required to support these associations and to explore the relationship between UGI involvement and specific racial and ethnic populations.

Several gaps were also evident across the systematic reviews. Some did not provide an overall prevalence synthesis, while others included pediatric or mixed-age populations, potentially contributing to higher estimates than those observed in adult-only cohorts, as UGI involvement has been reported more frequently in pediatric patients with CD.^135-137^ In contrast, Cohen et al. restricted inclusion to adults and reported a median prevalence across eligible studies.^10^

This scoping review represents one of the most comprehensive syntheses to date specifically focused on the epidemiologic characteristics of adult UGI-CD over the past 30 years. Data were compiled from 117 primary studies encompassing over 5000 patients with UGI-CD to understand the prevalence patterns and associated epidemiologic characteristics across diverse geographic and clinical contexts. The breadth of this analysis allows for a more complete appreciation of existing knowledge gaps and highlights key methodological inconsistencies that should be addressed in future research. However, this scoping review does have several limitations. First, reporting of UGI-CD across primary studies was inconsistent, with heterogeneous definitions, variable study designs, and incomplete demographic or site-level data, which complicates interpretation and limits cross-cohort comparability. As a result, we were unable to formally stratify prevalence estimates based on diagnostic approach (eg, routine vs symptom-driven endoscopy, use of histological confirmation, or imaging-based detection), as these details were not consistently reported. Some cohorts relied on population-based registries, chart review, or diagnostic practices such as CTE^103-105^^,^^107^^,^^111^^,^^119^^,^^120^^,^^123^ while others incorporated systematic upper endoscopy with routine biopsy, often yielding much higher estimates (≥40%).^2^^,^^34^^,^^60^^,^^62^^,^^114^ To reduce heterogeneity, we restricted inclusion to the past 30 years to better reflect contemporary diagnostic practices, and we excluded studies that classified L4 involvement beyond the jejunum to restrict analysis to the UGI tract as defined by the Montreal classification. Although some cohorts did not explicitly differentiate adult and pediatric patients, we prioritized adult-specific data whenever available. Finally, a formal meta-analysis was not conducted due to substantial methodological variability across studies, including heterogeneous definitions of UGI-CD, differences in study populations, and inconsistent diagnostic practices. Given that the primary objective of this scoping review was to map the breadth of epidemiologic reporting practices and identify sources of methodological heterogeneity rather than generate pooled effect estimates, formal risk-of-bias assessment was not incorporated into the review design, consistent with current scoping review methodology. Nonetheless, differences in study design, diagnostic intensity, ascertainment practices, and reporting quality likely contributed substantially to the variability in reported prevalence estimates and should be considered when interpreting the findings. The scoping design allowed us to comprehensively map the literature, identify important reporting gaps, and highlight methodological considerations that future prospective studies and systematic reviews should address.

A pertinent gap in the data was the reporting on demographic variables and modifiers specifically in the UGI-CD subgroup. Where reported, age and sex estimates were similar to those summarized by Cohen et al.^10^ However, age and sex were reported for the UGI-CD subgroup in only 16 and 24 of 117 studies, respectively. Smoking status, family history, and EIMs were reported in only 12, 11, and five studies, limiting conclusions about their relationship with UGI-CD. Although ethnicity was reported more frequently, comparative data remained limited. Understanding the prevalence of UGI-CD in specific ethnicities can help inform future screening guidelines and diagnostic practices for those populations who are most susceptible. This is especially important given the frequent asymptomatic nature of UGI-CD and association of L4 disease with downstream disease complications such as hospitalizations, stricturing, and need for surgery.^13^^,^^29^^,^^41^^,^^56-62^^,^^64^ Increased reporting of these parameters would help clarify whether UGI involvement is concentrated in particular demographic groups and determine if modifiers, such as smoking or family history, shape the likelihood or phenotype of UGI disease. It would also enable further exploration of potential prognostic implications, such as whether patients with UGI-CD are predisposed to more aggressive behavior, higher surgical risk, or distinct patterns of extraintestinal involvement. By mapping the literature, identifying reporting gaps, and highlighting the sources of heterogeneity across studies, this review highlights the methodological considerations needed in future cohorts for a robust synthesis.

## 5. Conclusion

This study highlights key sources of variability and important gaps in the epidemiology of adult UGI-CD that are not consistently addressed in existing literature. In particular, it provides an assessment of geographic and ethnic variation, demonstrates that isolated UGI-CD represents a small minority of cases, and identifies significant limitations in demographic reporting across studies that constrain interpretation of epidemiologic patterns. While the median prevalence of 8.8% (IQR 3.99%-14.5%) is broadly consistent with prior reports, estimates remain highly variable due to differences in definitions of UGI involvement. There may be ethnic and geographic variation in the prevalence of UGI-CD, and this is an area for further research. Existing literature also suggests that those with UGI involvement may have higher rates of penetrating and stricturing phenotypes, which tend to portend a higher risk of disease complications. Inconsistent reporting of UGI involvement in studies and variable diagnostic practices among clinicians remain key limitations in understanding the epidemiologic trends of UGI-CD. Future studies should apply existing consensus guidance on UGI-CD and use systematic diagnostic approaches and consistent reporting to clarify its epidemiology and improve recognition of this phenotype in adult CD. These recommendations should be refined as the evidence base evolves.

## Supplementary Material

jjag168_Supplementary_Data

## Data Availability

All data underlying this article are available within the manuscript and its supplementary material. No new data were generated beyond the published studies cited.
