# Supplementary material for: Prevalence and epidemiologic features of adult upper gastrointestinal Crohn’s disease over the last 30 years: a scoping review
Source: J Crohns Colitis. 2026 Oct 30;20(10):jjag168. doi: 10.1093/ecco-jcc/jjag168 (PMC13722441; doi:10.1093/ecco-jcc/jjag168)
Supplement: Supplementary file 1 [file jjag168_supplementary_data.zip › Supp fig 3 prevalence distribution figure.docx]

Studies including mixed geographic populations or multinational cohorts were not assigned to a single geographic category in the figure because regional attribution could not be reliably standardized.

Reported prevalence estimates may be influenced by study design, diagnostic intensity, use of systematic endoscopy, histologic confirmation, and ascertainment practices.

| **Geographic Area** | **Countries Included** | **References** |
| --- | --- | --- |
| South Asia | India, Sri Lanka, Malaysia | 21, 31, 46, 93, 116, 128 |
| East Asia | China, South Korea, Japan | 24, 26, 27, 34, 47, 48, 51, 54, 55, 62, 63, 91, 95, 105, 114 |
| Middle East | Saudi Arabia, Israel, Turkey | 3, 19, 49, 80, 111 |
| Europe | Italy, Germany, Portugal, Czech Republic, Switzerland, Romania, Netherlands, Hungary, Denmark, Norway, Poland | 23, 30, 35, 37, 42, 43, 50, 56, 59, 66, 74, 81, 83, 86, 109, 112, 119, 120, 121, 133 |
| North America | Canada, United States | 29, 40, 41, 57, 64, 82, 84, 88, 96, 97, 98, 99, 100, 101, 102, 103, 104, 106, 117, 129, 132 |
| South America | Brazil | 32, 77, 79, 125 |
