# Supplementary material for: Prevalence and epidemiologic features of adult upper gastrointestinal Crohn’s disease over the last 30 years: a scoping review
Source: J Crohns Colitis. 2026 Oct 30;20(10):jjag168. doi: 10.1093/ecco-jcc/jjag168 (PMC13722441; doi:10.1093/ecco-jcc/jjag168)
Supplement: Supplementary file 1 [file jjag168_supplementary_data.zip › SupplementaryFigure1_PrevalenceEpidemiologyUGICD.docx]

Supplementary Figure 1. Full electronic search strategy

1. Crohn*.ti,ab,kw,kf.

2. Crohn disease/ use medall,cctr

3. exp Crohn disease/ use oemezd

4. 1 or 2 or 3 [CD]

5. upper gastrointestinal tract/ or stomach/ or esophagus/ or duodenum/

6. (upper gastro* or gastroesophag* or gastro-esophag* or gastro-oesophag* or upper GI or UGI or esophag* or oesophag* or stomach or gastric* or duoden* or "L4").ti,ab,kw,kf.

7. (jejunum or jejunal).ti,ab,kw,kf.

8. 5 or 6 or 7 [UGIT]

9. 4 and 8 [CD and UGIT]

10. conference abstract.pt. or conference review.pt. use oemezd

11. 9 not 10

12. trial registry record.pt.

13. "clinicaltrials.gov".so.

14. 11 not (12 or 13)

15. case report/ or case reports/

16. ((case report or a rare case or a case of) not review).ti.

17. 14 not (15 or 16)

18. (exp animals/ or exp animal experimentation/ or exp animal experiment/ or exp models animal/ or nonhuman/ or exp vertebrate/ or exp vertebrates/) not (exp humans/ or exp human experimentation/ or exp human experiment/) [CADTH animal filter- Standard - MEDLINE, Embase. In: Search Filters Database. Ottawa: CADTH; 2024: https://searchfilters.cda-amc.ca/link/6. ]

19. 17 not 18

20. limit 19 to english language

21. remove duplicates from 20
