# Supplementary material for: Prevalence and epidemiologic features of adult upper gastrointestinal Crohn’s disease over the last 30 years: a scoping review
Source: J Crohns Colitis. 2026 Oct 30;20(10):jjag168. doi: 10.1093/ecco-jcc/jjag168 (PMC13722441; doi:10.1093/ecco-jcc/jjag168)
Supplement: Supplementary file 1 [file jjag168_supplementary_data.zip › SupplementaryFigure2_PrevalenceEpidemiologyUGICD.docx]

Supplementary Figure 2. Preferred reporting items for systematic reviews and meta-analyses (PRISMA) flow diagram of the study selection process.
